# Supplementary material for: Effect of vegetable oil on ovarian steroidogenesis- A transcriptome approach to understand molecular mechanisms of hypothalamus pituitary and gonad axis (HPG) in Ompok bimaculatus
Source: PLoS One. 2024 Dec 4;19(12):e0309311. doi: 10.1371/journal.pone.0309311 (PMC11616869; doi:10.1371/journal.pone.0309311)
Supplement: Attachment — Submitted filename: PONE-D-24-14273 (Editor).pdf [file pone.0309311.s002.pdf]

PLOS ONE

Effect of vegetable oil on ovarian steroidogenesis- A transcriptome approach to understand molecular mechanisms of hypothalamus pituitary and gonad axis in Ompok bimaculatus

--Manuscript Draft--

**Manuscript Number:**	PONE-D-24-14273
**Article Type:**	Research Article
**Full Title:**	Effect of vegetable oil on ovarian steroidogenesis- A transcriptome approach to understand molecular mechanisms of hypothalamus pituitary and gonad axis in Ompok bimaculatus
**Short Title:**	Effect of vegetable oil on ovarian steroidogenesis of Ompok bimaculatus
**Corresponding Author:**	Jitendra Kumar Sundaray, PhD<br>CIFA: Central Institute of Freshwater Aquaculture<br>Bhubaneswar, Odisha INDIA
**Keywords:**	Estradiol; Lipid; NGS; Testosterone; Vegetable oil
**Abstract:**	Ompok bimaculatus is a commercially important food fish of Northeast India. For higher production of this species, the molecular mechanisms underlying its reproductive physiology is an important aspect to study. Vegetable oils are rich in ω3 and ω6 fatty acids and fat-soluble vitamins, that enhance reproductive performances. In the present study, O. bimaculatus were first fed with vegetable oil at 3, 5, 7 and 9 % in the diet. The best treatment was determined by the analysis of relative fecundity, fertilisation rate, GSI and HSI. Comparative transcriptomics analysis of gonadal tissues between control and best treatment was done using Illumina NextSeq500. The bioinformatics analysis by Trinity v2.11.0 and edgeR v3.32.1 identified 36 DEGs related to reproduction, those were later validated by qPCR analysis. The DEGs revealed, regulation of HPG axis through retinoid signalling pathway, IGF pathway and folliculogenesis pathway, including inhibitory effects of dietary vegetable oil on fish gonadal maturation.
**Order of Authors:**	Sagar Chandra Mandal, PhD
	Partha Sarathi Tripathy, M.F.Sc. in Fish Genetics and Breeding
	Ananya Khatei, M.F.Sc. in Fish Genetics and Breeding
	Ningthoujam Chaoba Devi, M.F.Sc. in Fish Genetics and Breeding
	Pradyut Biswas, PhD
	Jitendra Kumar Sundaray, PhD
	Farhana Hoque, PhD
	Janmejay Parhi, PhD
**Opposed Reviewers:**	
**Additional Information:**	
**Question**	**Response**
**Financial Disclosure**<br><br>Enter a financial disclosure statement that describes the sources of funding for the work included in this submission. Review the submission guidelines for detailed requirements. View published research articles from *PLOS ONE* for specific examples.	The author(s) received no specific funding for this work.

Powered by Editorial Manager® and ProduXion Manager® from Aries Systems Corporation

This statement is required for submission and **will appear in the published article** if the submission is accepted. Please make sure it is accurate.<br><br>**Funded studies**<br>Enter a statement with the following details:<br>• Initials of the authors who received each award<br>• Grant numbers awarded to each author<br>• The full name of each funder<br>• URL of each funder website<br>• Did the sponsors or funders play any role in the study design, data collection and analysis, decision to publish, or preparation of the manuscript?<br><br>Did you receive funding for this work?	
**Competing Interests**<br><br>Use the instructions below to enter a competing interest statement for this submission. On behalf of all authors, disclose any competing interests that could be perceived to bias this work—acknowledging all financial support and any other relevant financial or non-financial competing interests.<br><br>This statement is required for submission and **will appear in the published article** if the submission is accepted. Please make sure it is accurate and that any funding sources listed in your Funding Information later in the submission form are also declared in your Financial Disclosure statement.<br><br>View published research articles from *PLOS ONE* for specific examples.	The authors have declared that no competing interests exist.

Powered by Editorial Manager® and ProduXion Manager® from Aries Systems Corporation

**NO authors have competing interests**<br><br>Enter: *The authors have declared that no competing interests exist.*<br><br>**Authors with competing interests**<br><br>Enter competing interest details beginning with this statement:<br><br>*I have read the journal's policy and the authors of this manuscript have the following competing interests: [insert competing interests here]*<br><br>* typeset	
**Ethics Statement**<br><br>Enter an ethics statement for this submission. This statement is required if the study involved:<br><br>• Human participants<br>• Human specimens or tissue<br>• Vertebrate animals or cephalopods<br>• Vertebrate embryos or tissues<br>• Field research<br><br>Write "N/A" if the submission does not require an ethics statement.<br><br>General guidance is provided below. Consult the submission guidelines for detailed instructions. **Make sure that all information entered here is included in the Methods section of the manuscript.**	All the fish specimens of O. bimaculatus used in this study were collected from the third generation of domesticated stock in the Wet Lab of College of Fisheries, CAU (Imphal), Tripura, India. Ethical approval, specimen collection and maintenance were performed strictly according to ethical standards of the Institute's Animal Ethics Committee, College of Fisheries, Central Agricultural University, Imphal, India (IAEC/COF-CAU No. A8/213422/2021).

Powered by Editorial Manager® and ProduXion Manager® from Aries Systems Corporation

**Format for specific study types**<br><br>**Human Subject Research (involving human participants and/or tissue)**<br>• Give the name of the institutional review board or ethics committee that approved the study<br>• Include the approval number and/or a statement indicating approval of this research<br>• Indicate the form of consent obtained (written/oral) or the reason that consent was not obtained (e.g. the data were analyzed anonymously)<br><br>**Animal Research (involving vertebrate animals, embryos or tissues)**<br>• Provide the name of the Institutional Animal Care and Use Committee (IACUC) or other relevant ethics board that reviewed the study protocol, and indicate whether they approved this research or granted a formal waiver of ethical approval<br>• Include an approval number if one was obtained<br>• If the study involved *non-human primates*, add *additional details* about animal welfare and steps taken to ameliorate suffering<br>• If anesthesia, euthanasia, or any kind of animal sacrifice is part of the study, include briefly which substances and/or methods were applied<br><br>**Field Research**<br><br>Include the following details if this study involves the collection of plant, animal, or other materials from a natural setting:<br>• Field permit number<br>• Name of the institution or relevant body that granted permission	
**Data Availability**<br><br>Authors are required to make all data underlying the findings described fully available, without restriction, and from the time of publication. PLOS allows rare exceptions to address legal and ethical concerns. See the PLOS Data Policy and FAQ for detailed information.	Yes - all data are fully available without restriction

Powered by Editorial Manager® and ProduXion Manager® from Aries Systems Corporation

A Data Availability Statement describing where the data can be found is required at submission. Your answers to this question constitute the Data Availability Statement and **will be published in the article**, if accepted.<br><br>**Important:** Stating 'data available on request from the author' is not sufficient. If your data are only available upon request, select 'No' for the first question and explain your exceptional situation in the text box.<br><br>Do the authors confirm that all data underlying the findings described in their manuscript are fully available without restriction?	
**Describe where the data may be found in full sentences. If you are copying our sample text, replace any instances of XXX with the appropriate details.**<br><br>• If the data are **held or will be held in a public repository**, include URLs, accession numbers or DOIs. If this information will only be available after acceptance, indicate this by ticking the box below. For example: *All XXX files are available from the XXX database (accession number(s) XXX, XXX.).*<br>• If the data are all contained **within the manuscript and/or Supporting Information files**, enter the following: *All relevant data are within the manuscript and its Supporting Information files.*<br>• If neither of these applies but you are able to provide **details of access elsewhere**, with or without limitations, please do so. For example:<br><br>*Data cannot be shared publicly because of [XXX]. Data are available from the XXX Institutional Data Access / Ethics Committee (contact via XXX) for researchers who meet the criteria for access to confidential data.*<br><br>*The data underlying the results presented in the study are available from (include the name of the third party*	Sequence reads are available from the NCBI sequence read archive (SRA) under the accession number PRJNA705413.

and contact information or URL).<br>• This text is appropriate if the data are owned by a third party and authors do not have permission to share the data.<br><br>* typeset	
Additional data availability information:	

Powered by Editorial Manager® and ProduXion Manager® from Aries Systems Corporation

Cover letter

April 08, 2024

Editorial Department

Dear Editor-in-chief,

We hereby submit a manuscript titled "**Effect of vegetable oil on ovarian steroidogenesis- A transcriptome approach to understand molecular mechanisms of hypothalamus pituitary and gonad axis in *Ompok bimaculatus*"** for consideration of publication in your esteemed journal "**Plos One**". We also affirm that the manuscript or part of the manuscript has not been published elsewhere and/or submitted simultaneously elsewhere for publication and this study does not involve human subjects. We are mentioning Email-IDs of authors of this publication as, Sagar Chandra Mandal (scmandal02@gmail.com), Janmejay Parhi (jjparhi@gmail.com), Ananya Khatei (ananya137@gmail.com), Ningthoujam Chaoba Devi (ningthoujamchaoba8@gmail.com), Partha Sarathi Tripathy (partha.s.tripathy@nord.no), Pradyut Biswas (pradyutbiswas@gmail.com), Farhana Hoque (farhanahoque22@gmail.com) and Jitendra Kumar Sundaray (jsundaray@gmail.com).

Lipids play significant roles as sources of metabolic energy for growth and reproduction in fishes. Large amounts of lipids are required both for egg production and breeding activities. During maturation and spawning, there is a need for high energy in the gonad. The lipid stored in the liver and muscle is transferred and move to the gonad. This is why in this study; molecular mechanisms of reproductive process have been analyzed in *O. bimaculatus* with dietary inclusion of vegetable oil. The reproductive genes of fish are huge in number and are associated with the processes that start from gonadal development to the release of milt and oocyte. So, to understand the molecular mechanism of dietary vegetable oil supplements on the reproductive process, comparative transcriptomics analysis has been carried out. The set of reproductive genes responsible for reproduction and spawning have been identified using OMICS approach and further validated by using qPCR. This study will provide a useful subject matter in the field of aquaculture genomics molecular biology and genetics, as well as development of commercial breeding programmes through proper feed development targeting to enhance production.

With best regards.

Yours Sincerely,

The corresponding author of the manuscript:

Dr. Jitendra Kumar Sundaray

Principal Scientist,

ICAR- Central Institute of Freshwater Aquaculture, Bhubaneswar- 751002, Odisha, India.

Ph: +919437166872; E-mail: jsundaray@gmail.com; https://orcid.org/0000-0001-9311-0636

Manuscript Click here to access/download;Manuscript;Final_Manuscript.docx

Title page

Effect of vegetable oil on ovarian steroidogenesis- A transcriptome approach to understand molecular mechanisms of hypothalamus pituitary and gonad axis in *Ompok bimaculatus*

Sagar Chandra Mandal[a], Partha Sarathi Tripathy[b, c], Ananya Khatei[b, d], Ningthoujam Chaoba Devi[a], Pradyut Biswas[a], Jitendra Kumar Sundaray[e, *], Farhana Hoque[e] and Janmejay Parhi[a, *]

[a] College of Fisheries, Central Agricultural University (Imphal), Lembucherra, Tripura-799210, India

[b] Faculty of Biosciences and Aquaculture, Nord University, 8026, Bodø, Norway

[c] Rani Lakshmibai Central Agricultural University, Jhansi- 284003, Uttar Pradesh, India

[d] Directorate of Coldwater Fisheries Research, Bhimtal-263136, Uttarakhand, India

[e] ICAR-Central Institute of Freshwater Aquaculture, Kausalyaganga, Bhubaneswar-751002, Odisha, India

* Jitendra Kumar Sundaray and Janmejay Parhi should be considered as joint corresponding author

Corresponding author:

Dr. Jitendra Kumar Sundaray

Principal Scientist,

ICAR-Central Institute of Freshwater Aquaculture, Bhubaneswar- 751002, Odisha, India.

Ph: +919437166872

E-mail: jsundaray@gmail.com; https://orcid.org/0000-0001-9311-0636

Abstract

Ompok bimaculatus is a commercially important food fish of Northeast India. For higher production of this species, the molecular mechanisms underlying its reproductive physiology is an important aspect to study. Vegetable oils are rich in ω3 and ω6 fatty acids and fat-soluble vitamins, that enhance reproductive performances. In the present study, *O. bimaculatus* were first fed with vegetable oil at 3, 5, 7 and 9 % in the diet. The best treatment was determined by the analysis of relative fecundity, fertilisation rate, GSI and HSI. Comparative transcriptomics analysis of gonadal tissues between control and best treatment was done using Illumina NextSeq500. The bioinformatics analysis by Trinity v2.11.0 and edgeR v3.32.1 identified 36 DEGs related to reproduction, those were later validated by qPCR analysis. The DEGs revealed, regulation of HPG axis through retinoid signalling pathway, IGF pathway and folliculogenesis pathway, including inhibitory effects of dietary vegetable oil on fish gonadal maturation.

Keywords: Estradiol; Lipid; NGS; Testosterone; Vegetable oil.

1. Introduction

Lipids play significant roles as sources of metabolic energy for growth and reproduction in fishes. Large amounts of lipids are required both for egg production and breeding activities (1, 2). During maturation and spawning, there is a need for high energy in the gonad. The lipid stored in the liver and muscle is transferred and move to the gonad (3). In adult fishes, different lipid classes in gonads, liver and muscle influence sexual maturation and spawning (4, 5). Essential fatty acids (EFA), particularly docosahexaenoic acid (DHA: 22:6n-3) and eicosapentaenoic acid (EPA: 20:5n-3), are one of the major nutritional factors significantly affecting reproductive performance in fish (6). Phospholipids are the primary lipid required during egg yolk development, embryonic and early larval development in fishes (7). Polyunsaturated fatty acids like arachidonic acid (AA:20:4n-6) is also considered as an essential compound because of their involvement in the production of eicosanoid (8), particularly prostaglandins, which are involved in several reproductive processes like the production of steroid hormones and gonadal development such as ovulation (9). Most fish cannot synthesise *n-3* and *n-6* PUFA *de novo* so, they must be supplied in the diet. High unsaturated fatty acid (HUFA) and polyunsaturated fatty acids (PUFA) in

brooder feed significantly affect fecundity, fertility, hatching, and viability of fish eggs and larval physiological fitness (10, 11).

Ompok bimaculatus (Pabda) is a highly demanded fish species in aquaculture, especially in the eastern, north-eastern India, and Bangladesh, because of its good taste and flavour (12). The Fish has been recognised as state fish of Tripura. Various efforts are made to improve brood stock quality to produce sufficient and good quality offspring for high production. In the present study, dietary inclusion of vegetable oil was evaluated to understand the effect on the molecular reproductive process. The reproductive genes of fish are huge in number and are associated with the processes that start from gonadal development to the release of milt and oocyte. The transcriptomic study usually targets the whole transcripts (RNA) transcribed from a given cell or tissue type from an organism at a particular time (13). Comparative transcriptomic provide information on the differential expression pattern of important genes between treated and control groups. So to understand the molecular mechanism of dietary vegetable oil supplements on the reproductive process, comparative transcriptomics analysis was carried out. The set of reproductive genes responsible for reproduction and spawning are identified using OMICS approach and further validated by using qPCR.

2. Materials and methods

2.1. Ethical statement

All the fish specimens of *O. bimaculatus* used in this study were collected from the third generation of domesticated stock in the Wet Lab of College of Fisheries, CAU (Imphal), Tripura, India. Ethical approval, specimen collection and maintenance were performed strictly according to ethical standards of the Institute's Animal Ethics Committee, College of Fisheries, Central Agricultural University, Imphal, India (IAEC/COF-CAU No. A8/213422/2021).

2.2. Experimental setup

The third generation of the domesticated stock of *O. bimaculatus* was kept in circular tanks (12 feet diameter, 4 feet depth, 2000 litre water) for six months in triplicates, i.e., three tanks each in control and treatment groups. The stocking density was kept at 30 fish per tank, and proper water quality parameters were maintained to minimise environmental effects on the experiment. The control group were fed with an

artificially formulated diet without adding any vegetable oil (Table 1). The treated group were fed a diet with the inclusion of vegetable oil at 3, 5, 7 and 9 % for T1, T2, T3 and T4, respectively (Table 1). The crude protein content was 30-35% in each feed. The best treatment among these, i.e., T2, was taken for transcriptomics analysis. Moreover, the relative fecundity, fertilisation rate, GSI (Gonado Somatic Index), HSI (Hepato Somatic Index), etc., were calculated based on the methods of earlier published work (14). These parameters were calculated using ten fish specimens from each replicate.

2.3. Sample collection and RNA extraction

The ovary tissues were collected in triplicates from each group (control and T2) and kept at -20°C for qPCR analysis. The tissue samples were pooled from the control and treated group (T2) and were marked as PCO and PTO, respectively, for transcriptomics analysis. The tissue samples were kept in liquid nitrogen until RNA isolation. The total RNA was isolated by RNeasy Plus Universal Mini Kit (Qiagen, USA), as per the manufacturer's protocol. The quality and quantity of the isolated RNA were checked using 1% denaturing RNA agarose gel and NanoDrop, respectively.

2.4. Library preparation and sequencing

Library preparation was performed with Illumina TruSeq Stranded mRNA Sample Prep kit (Illumina, USA) according to the standard protocol set from the manufacturer. The RNA sample concentration used from both samples were 1355 ng/µl. The library quality was tested using Agilent 4200 Tape Station system (Agilent Technologies, USA). The Paired-end (PE) Illumina libraries were loaded onto NextSeq500 for cluster generation and sequencing using 2 x 75 bp chemistry. This PE sequencing allows the template fragments to be sequenced in both forward and reverse directions. The illumine kit reagents were used in the binding of samples to complementary adapter oligos on the PE flow cell. The adapters were designed to allow selective cleavage of the forward strands after re-synthesis of the reverse strand during sequencing. The copied reverse strand was used to sequence from the opposite end of the fragment.

2.5. de novo assembly and DE analysis

The raw reads generated in fastq format were then taken for quality checking by FastQC v0.11.9 (15). The low-quality reads (phred score < 30), Illumina adapters and any required overrepresented

sequences for each sample were removed by quality trimming using Cutadapt v3.2 (16). Sequence reads are available from the NCBI sequence read archive (SRA) under the accession number PRJNA705413. Trimmed reads were again checked for quality using FastQC v0.11.9 (15). The final quality reads of all the samples were concatenated to a single file and loaded onto Trinity software package v2.11.0 (17) with default parameters to assemble high-quality reads into contigs. The Trinity package helped to reconstruct the transcripts and isoforms into their final form. The transcriptome assembly quality was checked using sanger-pathogens/assembly-stats v1.0.1 to generate standard metrics and N50 statistics. Mapping and abundance estimation for each sample were done from the align_and_estimate_abundance.pl utility in Trinity (17) with default parameters and RSEM as abundance estimation method. The count tables generated for each sample were merged for further differential expression (DE) analysis using Trinity's abundance_estimates_to_matrix.pl utility (17). The merged RSEM isoform count matrix was also used for calculating fold changes between PCO and PTO by edgeR v3.32.1 (18) package in R. Additionally, Fragments Per Kilobase of Transcript per Million Mapped (FPKM) values from each sample were done using run_TMM_normalization_write_FPKM_matrix.pl function in Trinity (17). Additionally, the percentage of reads assembled were computed using bowtie2 v2.4.2 (19), and the completeness of the assembly was checked using BUSCO v5.0.0 (20) with the "Actinopterygii" lineage dataset i.e. actinopterygii_odb10 (2021-02-19). The BUSCO was run in the transcriptome assembly mode.

2.6. Functional annotation and CDS prediction of differentially expressed transcript

The coding transcripts from the *de novo* assembled transcripts were detected using TransDecoder v5.5.0. Then the functional annotation of the CDS was performed using DIAMOND (BLASTX alignment mode) v2.0.7 (21) to find the homologous sequences for the CDS against NR (non-redundant protein database) from NCBI. Reads of PCO and PTO were pooled to identify the CDS of each sample, and the reads from each of the samples were mapped on the final set of pooled CDS using BWA-MEM toolkit v0.7.17 (22). The read count (RC) values were calculated from the resulting mapping, and those CDS with at least 85% coverage and 3X read depth were considered for downstream analysis for each sample. These FPKM values of the top 50 differentially expressed genes (DEGs) and reproductive genes were used for

heatmap generation between PCO and PTO using gdata v2.18.0 (23) and pheatmap v1.0.12 (24) package in R. Among the top 50 DEGs, 25 were taken as up-regulated and 25 as down-regulated. Log2 Fold Change was calculated for further analysis of differential expression of each transcript between PCO and PTO by taking the log of PTO FPKM vs PCO FPKM value. All the DEGs with p-value <0.05 and log2 fold changes were taken for volcano plot generation in gdata v2.18.0 (23) and gplots v3.1.1 (25) package in R.

2.7. Gene ontology (GO) and KEGG pathway mapping

The DEGs were taken for GO and KEGG pathway determination on shinyGO v0.61 (26), taking a p-value cut-off of 0.05. All the genes were analysed against three GO categories, i.e., Biological Process, Cellular Component and Molecular Function. The output of shinyGO analysis was plotted in the WEGO plot (27).

2.8. qPCR validation

The QuantStudio™ 5 Real-Time PCR (Applied Biosystems, USA), based on SYBR green chemistry, was used to validate candidate genes related to reproduction. The gene-specific primers were designed based on transcriptomics data. The gonad tissues were sampled from both PCO and PTO in triplicates. Total RNA was isolated from all the tissues, using Quick-RNA Miniprep Plus Kit (Zymo Research, USA) and cDNA was prepared, using iScript™ cDNA Synthesis Kit (Bio-Rad, USA) as per the manufacturer's protocol. qPCR reaction was performed by adding 5 µl of SYBR green master mix (Thermo Scientific, USA), forward and reverse gene-specific primers 1 µl each (1 pmol/µl), 1 µl of template cDNA (1 µg/µl) and nuclease-free water to make it up to a total volume of 10 µl. The. β-actin was taken as an internal control for expression level normalisation. Three technical replicates were made for each sample during qPCR. The analysis was done by the method of Pfaffl (28).

3. Results

3.1. Wet-lab experiment

The fertilisation rate and GSI were found to be highest for the T2 group of fishes i.e. averaging 80.02 and 23.83, respectively (Table 2). Interestingly, the HSI was found to be the lowest for T2 i.e. averaging 0.65 (Table 2). The relative fecundity was found to be highest for T2 and T3 groups, averaging

756.66 eggs/g body weight. From all the calculations, the T2 group was the best treatment, and no mortality was seen in any experimental group except T3.

3.2. RNA-Seq data analysis

The data generated for PCO and PTO were found to be 6.37 and 4.56 Gb, respectively. The total number of reads for PCO and PTO were found to be 42,479,658 and 30,366,474, respectively. The Trinity analysis generated 150,836 pooled transcripts with maximum and minimum transcript lengths of 16,683 and 201, respectively. The completeness of the BUSCO analysis on the present transcriptomic dataset was found to be 81%. Out of the total 2950 complete BUSCOs (C), 2488 complete and single-copy BUSCOs (S) and 462 complete and duplicated BUSCOs (D) were found. Moreover, 75 Fragmented BUSCOs (F) and 615 missing BUSCOs (M) were found in 3640 total BUSCO groups searched from the actinopterygii_odb10 dataset. The GC content was 47.24%. In total, 99.28% of the reads were assembled.

The present transcriptomics showed maximum BLAST hits with *Pangasianodon hypophthalamus*, *Ictalurus punctatus* and *Tachysurus fulvidraco*. The total number of CDS for PCO and PTO were found to be 18,421 and 17,830, respectively. The volcano plot for all the DEGs in the dataset has been shown in Fig. 1. The heatmap for the top 50 DEGs has been shown in Fig. 2. The GO analysis for the DEGs (WEGO plot) has been shown for PCO and PTO in Fig 3a and 3b, respectively. We found candidate genes related to reproduction among the top 50 DEGs. But in addition to this we found other genes related to reproduction in the transcriptomics data, which were differentially expressed.

3.3. qPCR analysis

The qPCR validation of candidate genes related to reproduction supported the differential expression patterns analysed in the transcriptomics data. The *stard3, SF1-A, cyp19a1a, rxrba, rxrbb, rxrga, egr2a, cyp17a1, hsd3b*, etc. were differentially expressed in the gonad tissues. The *aqp3a* and *cyp17a1* were found to be the top down-regulated gene. Similarly, *hsd3b*, *ar* and *hsp70.1* were found to be the top up-regulated gene in T2. The average of technical replicates for each biological replicates have been shown in the present study. The differential expressions are shown as log2 fold changes than control in Table 3.

4. Discussion

The present study depicts the effect of vegetable oil on the female reproductive process at a molecular level in *O. bimaculatus*. Several studies earlier describe the beneficial effects of dietary lipid on ovarian development (29-32). Vegetable oils are rich sources of w3 and w6 fatty acids and are also fortified with vitamin A, D, E, and K. Reproductive indices analysed in this study, also gave a better result for the vegetable oil treated fishes. The GSI was found to be very high compared to the control group of fishes which suggests that the contents of the vegetable oil were efficient in enhancing ovarian development. The relative fecundity was very high compared to the control group of fishes, which suggests that the vegetable oil improved the number and quality of the oocytes.

Another important parameter was also measured, which indicates the ovarian status, i.e. the HSI. The hepatosomatic index reflects upon the developmental process of oocytes since vitellogenesis occurs in the liver. In most of the teleosts, the HSI is more during the initial ripening of the ovary and is decreased towards the final maturation (33). The reason behind this is that the liver is completely involved in carrying out vitellogenesis during the initial phase, and most of the stored energy is diverted towards ovarian development as the stages proceed (33). The HSI was found to be decreased significantly in the treated group of fishes, on an average 0.15, 0.29, 0.28 and 0.21 lower than control in T1, T2, T3 and T4, respectively. This indicates better ovarian development.

Comparative transcriptomics was done to analyse the differences between the fish fed with regular diet and fish fed with vegetable oil-enriched diet at a molecular level. Several essential genes that participate in ovarian development and vitellogenesis were revealed. These genes were also found to be showing similar differential expression pattern in qPCR analysis like that of NGS data. The regulations of these genes supported reproductive indices obtained and discussed above.

STAR gene (*stard3*) is the initiator of ovarian steroidogenesis in fish (34). The star protein transports cholesterol to the inner mitochondrial membrane, where steroidogenesis begins with converting cholesterol to pregnelone. The *stard3* gene was up-regulated in the fishes fed with vegetable oil compared to the control group of fishes. This gene is also among the top 50 differentially expressed genes in the present transcriptomics data. Vegetable oils contain a higher proportion of linoleic acid, which enhances

steroidogenesis in vertebrates (35). Vegetable oil contains a high amount of palmitic acid precursor of palmitoylethanolamide (PEA) (36). This PEA is responsible for the synthesis of star protein. This might be the reason for the increase in the production of star protein. Another essential gene that regulates *stard3* is the steroidogenic factor 1 (*SF1-A*). This gene was also found to be up-regulated in the treatment group of fish.

Vegetable oils have a higher amount of linoleic acid that enhances ovarian physiology. In the present study, the *cyp19a1* gene was found to be up-regulated. In some fishes, down-regulation of this gene disrupts the ovarian process and leads to testicular development (37). The *cyp19a1* shows stage-specific expressions in zebrafish and has been found abundantly in the follicular layer of vitellogenic oocytes (38). This suggests that *cyp19a1* plays a significant role in vitellogenesis and its up-regulation depicts its role in enhancing the vitellogenesis process.

Genes of the retinoid signalling pathways (*rxrba, rxrbb, rxrga*) showed up-regulation in the treatment group of fishes HPG axis is regulated through the retinoic acid signalling pathway. In females of higher vertebrates, retinoic acid signalling plays a major role in folliculognesis and affects the number and quality of oocytes (39). Vegetable oils are fortified with vitamin A, which is the source of retinoic acid. This retinoic acid enhances the retinoid signalling pathway, thereby improving the female reproductive performance. It has been found in previous studies that *hsd17b*, which is an essential factor in ovarian steroidogenesis, is positively regulated through the retinoic acid signalling pathway (40). Higher production of *hsd17b2* is induced by retinoic acid. In the present analysis, *hsd17b2* was up-regulated. This gene is responsible for the interconversion of Estrone (E1) to Estradiol (E2) (41). In the present study, 17β-estradiol receptor (*esr1*) was down-regulated, suggesting that E1 is getting converted to E2. E2 is the main regulatory factor for vitellogenesis and the final maturation of the oocyte. Although E2 is produced by the conversion of both testosterone and E1, the major pathway traversed its production is the E1 signalling pathway rather than the testosterone (42). The E2 binds to globulin in the blood and traverses to the liver for vitellogenesis. The down-regulation of *esr1* in the treated fishes also suggests that, its expression is stage and site specific in ovary.

The classical pathway in steroidogenesis involves the breakdown of cholesterol into pregnelone, after which the hydroxylation of pregnelone results in the formation of 17α-OH-progesterone. This involves *cyp17a1* and *hsd3b*, both of which were up-regulated in the present study. The conversion into 17α-OH-progesterone into 17α-20β-dihydroxy-4-pregnen-3-one requires *hsd20b*, which was found to be up-regulated in the treatment group of fishes. Other vital genes involved in enhancing oocyte maturation, such as *egf* and *tgfa,* were also found up-regulated in the treatment group of fishes, which suggests that vegetable oil enhances the process of vitellogenesis and accelerates final oocyte maturation (43).

Interestingly, the cyclin-dependent kinase family genes mainly were found up-regulated in the present study. The highly up-regulated *cdk2*, also known as active MPF (maturation promoting factor), indicates that the incorporation of vegetable oil enhanced the maturation process of the ovary. The CDKs are primarily associated with cell differentiation and proliferation, which also holds true for germ cells (44). Since the oocytes are in developing stages, CDKs (*cdk1, cdk2, cdk13, cdk15* and *cdk16*) were up-regulated in the present study.

During the folliculogenesis of the oocytes, testosterone and 11-ketotestosterone, which are primarily associated with male steroidogenesis, also have important roles. These two androgens express in a stage-specific manner in the ovary and stimulate cortical alveoli production (45). Therefore, androgen receptors show increased expression in the ovary during folliculogenesis. It has been studied that androgen receptor antagonist, when administered, induces early follicular atresia and inhibits growth responses in oocytes (46). The progesterone receptor was found to be up-regulated in the present experiment. This indicates that increased intake of dietary lipids enhanced the expression of both androgen and progesterone receptors, thereby aiding ovarian development.

A higher amount of linoleic acid in the vegetable oils enhanced ovarian steroidogenesis. Treating with linoleic acid increases the expression of insulin-like growth factors (IGFs) in higher vertebrates (47). In the present study, the insulin-like growth factors (1,2 and 3) were also up-regulated significantly. The *igf1* and *igf2* are found in the liver and gonads of vertebrates. However, *igf3* is known to be found only in fish and is gonad-specific. The *igf1* promotes oocyte maturation and proliferation, whereas *igf2* has a more

specific role in folliculogenesis and exhibits germinal vesicle breakdown (GVBD). The *igf3* also has similar functions in teleosts. In some fishes like the killifish, it has been found that IGFs are more potent in stimulating oocyte maturation than 17a-20b-dihydroxy-4-pregnen-3-one (MIS) (48).

In the present study specific genes, responsible for the inhibition of oocyte maturation are significantly down-regulated. These genes include the follistatin group of genes (*fsta*). Follistatin forms an important regulatory member of the *egf* and *tgfa* pathway (49). As discussed earlier, *egf* accelerates oocyte maturation. Follistatin could block the *egf* pathway and can hence oocyte maturation inhibitor. Since the treated group of fishes show significant down-regulation in the *fsta* gene, it could be suggested that incorporation of vegetable oil enhances the factors responsible for the enhancement of oocyte maturation and puts a check on the inhibitory factors. As mentioned earlier, *tgfa* is responsible for the oocyte maturation process. However, *tgfb* inhibits oocyte maturation (50).

Another important gene related to reproduction is the *hsp70* binding protein. Since *hsp70* is found in the gonads, it cannot be ignored that it modulates reproductive stress. In higher vertebrates, it has been found that *igf1* and *fsh* can reduce the expression of *hsp70* and reduce thermal and nutritional stress effects on the ovary (51), which might be the reason behind the down-regulation of *hsp70* in the treatment group of fish. Since the expression of both *igf1* and *hsp70* were significantly higher in the present study, it can be stated that there is little or no reproductive stress. Similarly, RAS-like estrogen-regulated growth inhibitor (*rerg*) that inhibits cellular proliferation was significantly down-regulated in the treatment group of fish. There is a significant down-regulation of the EGR group of genes (*egr1* and *egr2a*). These genes are highly expressed during stress, apoptotic signalling and tissue damage (52, 53).

The above findings suggest that incorporating fortified vegetable oils can prove highly beneficial for oocyte maturation and ovarian development. It improves ovarian steroidogenesis at a molecular level and maintains ovarian physiological processes.

Declaration of competing interest

The authors declare no conflict of interest.

CRediT authorship contribution statement

Sagar Chandra Mandal: Resources, Formal analysis, Investigation, Funding acquisition. **Ananya Khatei:** Visualisation, Software, Data curation, Writing- Original draft. **Ningthoujam Chaoba Devi:** Methodology, Data curation. **Partha Sarathi Tripathy:** Investigation, Validation, Data curation, Writing- Original draft. **Pradyut Biswas:** Resources, Formal analysis, Software. **Jitendra Kumar Sundaray:** Funding acquisition, Supervision, Writing- Review & editing. **Farhana Hoque:** Methodology, Software. **Janmejay Parhi:** Supervision, Investigation, Writing- Review & editing.

Acknowledgements

The authors are thankful to the Vice Chancellor, Central Agricultural University, Imphal, India, for providing infrastructure facilities to carry out this research. The authors thank the DBT-Twinning Pabda Project, Department of Biotechnology, Government of India (BT/PR25141/NER/95/1039/2017) for providing facilities and financial support to conduct the experiment.

References

1. Ebrahimnezhadarabi M, Saad C, Harmin S, Satar MA, Kenari A. Effects of Phospholipids in Diet on Growth of Sturgeon Fish {Huso-huso) Juveniles. Journal of Fisheries and Aquatic Science. 2011;6(3):247.

2. Tocher DR. Metabolism and functions of lipids and fatty acids in teleost fish. Reviews in fisheries science. 2003;11(2):107-84.

3. Singh R, Singh A, Tripathi M. Melatonin induced changes in specific growth rate, gonadal maturity, lipid and protein production in Nile tilapia Oreochromis niloticus (Linnaeus 1758). Asian-Australasian journal of animal sciences. 2012;25(1):37.

4. Huynh MD, Kitts DD, Hu C, Trites AW. Comparison of fatty acid profiles of spawning and non-spawning Pacific herring, Clupea harengus pallasi. Comparative Biochemistry and Physiology Part B: Biochemistry and Molecular Biology. 2007;146(4):504-11.

5. Mourente G, Megina C, Díaz-Salvago E. Lipids in female northern bluefin tuna (Thunnus thynnus thynnus L.) during sexual maturation. Fish Physiology and Biochemistry. 2001;24(4):351-63.

6. Watanabe T, Arakawa T, Kitajima C, Fujita S. Effect of nutritional quality of broodstock diets on reproduction of red sea bream. Nippon Suisan Gakkaishi. 1984;50(3):495-501.

7. Tocher DR. Glycerophospholipid metabolism. Biochemistry and molecular biology of fishes. 4: Elsevier; 1995. p. 119-57.

8. Farndale BM, Bell JG, Bruce MP, Bromage NR, Oyen F, Zanuy S, Sargent JR. Dietary lipid composition affects blood leucocyte fatty acid compositions and plasma eicosanoid concentrations in European sea bass (Dicentrarchus labrax). Aquaculture. 1999;179(1-4):335-50.
9. Moore A, Olsén KH, Lower N, Kindahl H. The role of F-series prostaglandins as reproductive priming pheromones in the brown trout. Journal of Fish Biology. 2002;60(3):613-24.
10. Fernández-Palacios H, Izquierdo MS, Robaina L, Valencia A, Salhi M, Vergara J. Effect of n− 3 HUFA level in broodstock diets on egg quality of gilthead sea bream (Sparus aurata L.). Aquaculture. 1995;132(3-4):325-37.
11. Izquierdo M, Tandler A, Salhi M, Kolkovski S. Influence of dietary polar lipids' quantity and quality of ingestion and assimilation of labelled fatty acids by larval gilthead seabream. Aquaculture Nutrition. 2001;7(3):153-60.
12. Parween S. Ompok bimaculatus (Bloch, 1797). In: Siddiqui KU, Islam MA, Kabir SMH, Ahmed M, Ahmed ATA, Rahman AKA, et al., editors. Encyclopedia of Flora and Fauna of Bangladesh. 23. Dhaka: Asiatic Society of Bangladesh; 2007. p. 127.
13. Wang Z, Gerstein M, Snyder M. RNA-Seq: a revolutionary tool for transcriptomics. Nature reviews genetics. 2009;10(1):57-63.
14. Ferosekhan S, Sahoo S, Giri S, Das B, Pillai B, Das P. Broodstock development, captive breeding and seed production of bagrid catfish, Mahanadi rita, Rita chrysea (Day, 1877). Aquaculture. 2019;503:339-46.
15. Andrews S. FastQC: a quality control tool for high throughput sequence data. Babraham Bioinformatics, Babraham Institute, Cambridge, United Kingdom; 2010.
16. Martin M. Cutadapt removes adapter sequences from high-throughput sequencing reads. EMBnet journal. 2011;17(1):10-2.
17. Grabherr MG, Haas BJ, Yassour M, Levin JZ, Thompson DA, Amit I, et al. Trinity: reconstructing a full-length transcriptome without a genome from RNA-Seq data. Nature biotechnology. 2011;29(7):644.
18. Robinson MD, McCarthy DJ, Smyth GK. edgeR: a Bioconductor package for differential expression analysis of digital gene expression data. Bioinformatics. 2010;26(1):139-40.
19. Langmead B, Salzberg SL. Fast gapped-read alignment with Bowtie 2. Nature methods. 2012;9(4):357.
20. Seppey M, Manni M, Zdobnov EM. BUSCO: assessing genome assembly and annotation completeness. Gene prediction: Springer; 2019. p. 227-45.
21. Buchfink B, Xie C, Huson DH. Fast and sensitive protein alignment using DIAMOND. Nature methods. 2015;12(1):59-60.
22. Li H. Aligning sequence reads, clone sequences and assembly contigs with BWA-MEM. arXiv preprint arXiv:13033997. 2013.

23. Warnes GR, Bolker B, Gorjanc G, Grothendieck G, Korosec A, Lumley T, et al. gdata: Various R programming tools for data manipulation. R package version. 2014;2(3):35.

24. Kolde R, Kolde MR. Package 'pheatmap'. R package. 2015;1(7):790.

25. Warnes GR, Bolker B, Bonebakker L, Gentleman R, Huber W, Liaw A, et al. gplots: Various R programming tools for plotting data. R package version. 2009;2(4):1.

26. Ge SX, Jung D, Yao R. ShinyGO: a graphical gene-set enrichment tool for animals and plants. Bioinformatics. 2020;36(8):2628-9.

27. Ye J, Zhang Y, Cui H, Liu J, Wu Y, Cheng Y, et al. WEGO 2.0: a web tool for analyzing and plotting GO annotations, 2018 update. Nucleic acids research. 2018;46(W1):W71-W5.

28. Pfaffl MW. A new mathematical model for relative quantification in real-time RT–PCR. Nucleic acids research. 2001;29(9):e45-e.

29. Lane RL, Kohler CC. Effects of dietary lipid and fatty acids on white bass reproductive performance, egg hatchability, and overall quality of progeny. North American Journal of Aquaculture. 2006;68(2):141-50.

30. Li L, Wang W, Yusuf A, Zhu Y, Zhou Y, Ji P, Huang X. Effects of dietary lipid levels on the growth, fatty acid profile and fecundity in the oriental river prawn, Macrobrachium nipponense. Aquaculture Research. 2020;51(5):1893-902.

31. Liu L, Chen L, Li K, Zhou Y, Li E. Effects of dietary lipid sources on ovary development and reproduction performance of female Eriocheir sinensis. Journal of Fishery Sciences of China. 2007;14:786-93.

32. Mattos R, Staples CR, Thatcher WW. Effects of dietary fatty acids on reproduction in ruminants. Reviews of reproduction. 2000;5(1):38-45.

33. Hismayasari IB, Marhendra APW, Rahayu S, Saidin SD, Supriyadi D. Gonadosomatic index (GSI), hepatosomatic index (HSI) and proportion of oocytes stadia as an indicator of rainbowfish Melanotaenia boesemani spawning season. International Journal of Fisheries and Aquatic Studies. 2015;2(5):359-62.

34. King SR, Stocco DM. Steroidogenic acute regulatory protein expression in the central nervous system. Frontiers in endocrinology. 2011;2:72.

35. Hughes J, Kwong WY, Li D, Salter AM, Lea RG, Sinclair KD. Effects of omega-3 and-6 polyunsaturated fatty acids on ovine follicular cell steroidogenesis, embryo development and molecular markers of fatty acid metabolism. Reproduction (Cambridge, England). 2010;141(1):105-18.

36. Keppel Hesselink J, de Boer T, Witkamp RF. Palmitoylethanolamide: a natural body-own anti-inflammatory agent, effective and safe against influenza and common cold. International journal of inflammation. 2013;2013.

37. Dong W, Willett KL. Local expression of CYP19A1 and CYP19A2 in developing and adult killifish (Fundulus heteroclitus). General and comparative endocrinology. 2008;155(2):307-17.
38. Goto-Kazeto R, Kight KE, Zohar Y, Place AR, Trant JM. Localization and expression of aromatase mRNA in adult zebrafish. General and comparative endocrinology. 2004;139(1):72-84.
39. Damdimopoulou P, Chiang C, Flaws J. Retinoic acid signaling in ovarian folliculogenesis and steroidogenesis. Reproductive Toxicology. 2019;87:32-41.
40. Suzuki H, Ozaki Y, Gen K, Kazeto Y. Japanese eel retinol dehydrogenases 11/12-like are 17-ketosteroid reductases involved in sex steroid synthesis. General and Comparative Endocrinology. 2021;305:113685.
41. Loux S, Conley A, Scoggin K, Ali HE-S, Dini P, Ball B. New insights in equine steroidogenesis: an in-depth look at steroid signaling in the placenta. Reproduction. 2020;160(1):65-82.
42. Ohta K, Yamaguchi S, Yamaguchi A, Okuzawa K, Gen K, Kagawa H, Matsuyama M. Biosynthesis of estradiol-17β in the ovarian follicles of the red seabream Pagrus major during vitellogenesis. Fisheries science. 2002;68(3):680-7.
43. Van Der Kraak G, Lister AL. The inhibitory control of oocyte maturation in the zebrafish (Danio rerio): the role of the G protein-coupled estrogen receptor and epidermal growth factor. Biology of reproduction. 2011;85(1):6-8.
44. Nagahama Y, Yamashita M. Regulation of oocyte maturation in fish. Development, growth & differentiation. 2008;50:S195-S219.
45. Forsgren KL, Young G. Stage-specific effects of androgens and estradiol-17beta on the development of late primary and early secondary ovarian follicles of coho salmon (Oncorhynchus kisutch) in vitro. Biology of Reproduction. 2012;87(3):64, 1-14.
46. Corriero A, Zupa R, Mylonas CC, Passantino L. Atresia of ovarian follicles in fishes, and implications and uses in aquaculture and fisheries. Journal of Fish Diseases. 2021.
47. Csillik Z, Faigl V, Keresztes M, Galamb E, Hammon H, Tröscher A, et al. Effect of pre-and postpartum supplementation with lipid-encapsulated conjugated linoleic acid on reproductive performance and the growth hormone–insulin-like growth factor-I axis in multiparous high-producing dairy cows. Journal of dairy science. 2017;100(7):5888-98.
48. Negatu Z, Hsiao S, Wallace R. Effects of insulin-like growth factor-I on final oocyte maturation and steroid production in Fundulus heteroclitus. Fish Physiology and Biochemistry. 1998;19(1):13-21.
49. Pang Y, Ge W. Epidermal growth factor and TGFα promote zebrafish oocyte maturation in vitro: potential role of the ovarian activin regulatory system. Endocrinology. 2002;143(1):47-54.
50. Li M-H, Niu M-H, Feng Y-Q, Zhang S-E, Tang S-W, Wang J-J, et al. Establishment of lncRNA-mRNA network in bovine oocyte between germinal vesicle and metaphase II stage. Gene. 2021;791:145716.

51. Sirotkin AV, Bauer M. Heat shock proteins in porcine ovary: synthesis, accumulation and regulation by stress and hormones. Cell Stress and Chaperones. 2011;16(4):379-87.
52. Decker EL, Skerka C, Zipfel PF. The early growth response protein (EGR-1) regulates interleukin-2 transcription by synergistic interaction with the nuclear factor of activated T cells. Journal of Biological Chemistry. 1998;273(41):26923-30.
53. Taefehshokr S, Key YA, Khakpour M, Dadebighlu P, Oveisi A. Early growth response 2 and Egr3 are unique regulators in immune system. Central-European journal of immunology. 2017;42(2):205.

Figure legends

Fig.1. Volcano plot showing top up and down-regulated genes. Up-regulated and down-regulated genes are marked with red and blue dots, respectively. The logFC and -$\log_{10}$ adjusted p-values were plotted using R.

Fig.2. Heatmap showing top 50 up and down-regulated genes. FPKM values of the top 50 DEGs and reproductive genes were used for heatmap generation between PCO and PTO using R.

Fig. 3. Gene ontology enrichment analysis of DEGs for **a.** PCO and **b.** PTO. DEGs described were analyzed for enrichment in three GO categories i.e. biological process, cellular component, and molecular function.

Figure 1

Click here to access/download;Figure;Fig1.tif

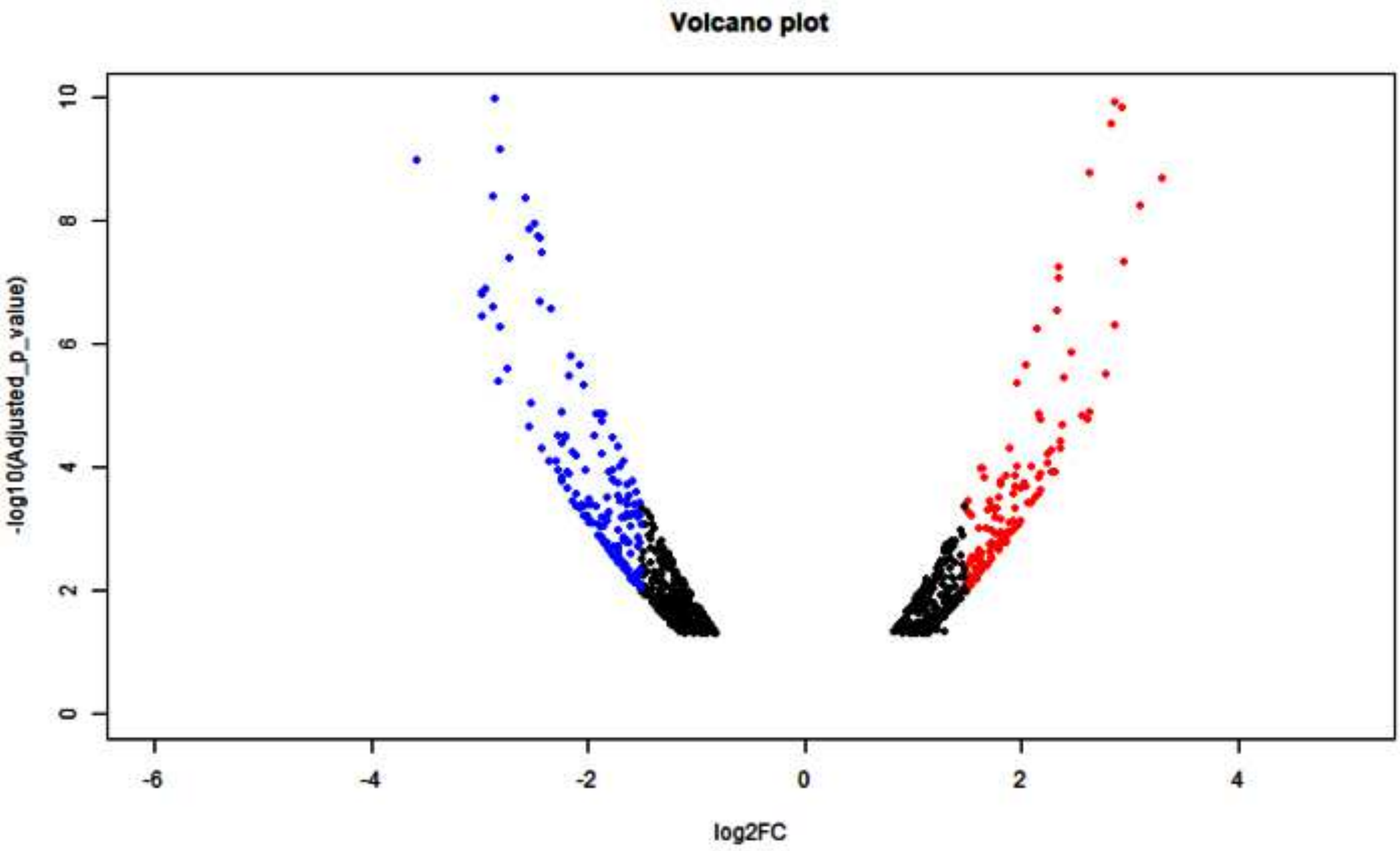

Figure 2

Click here to access/download;Figure;Fig2.tiff

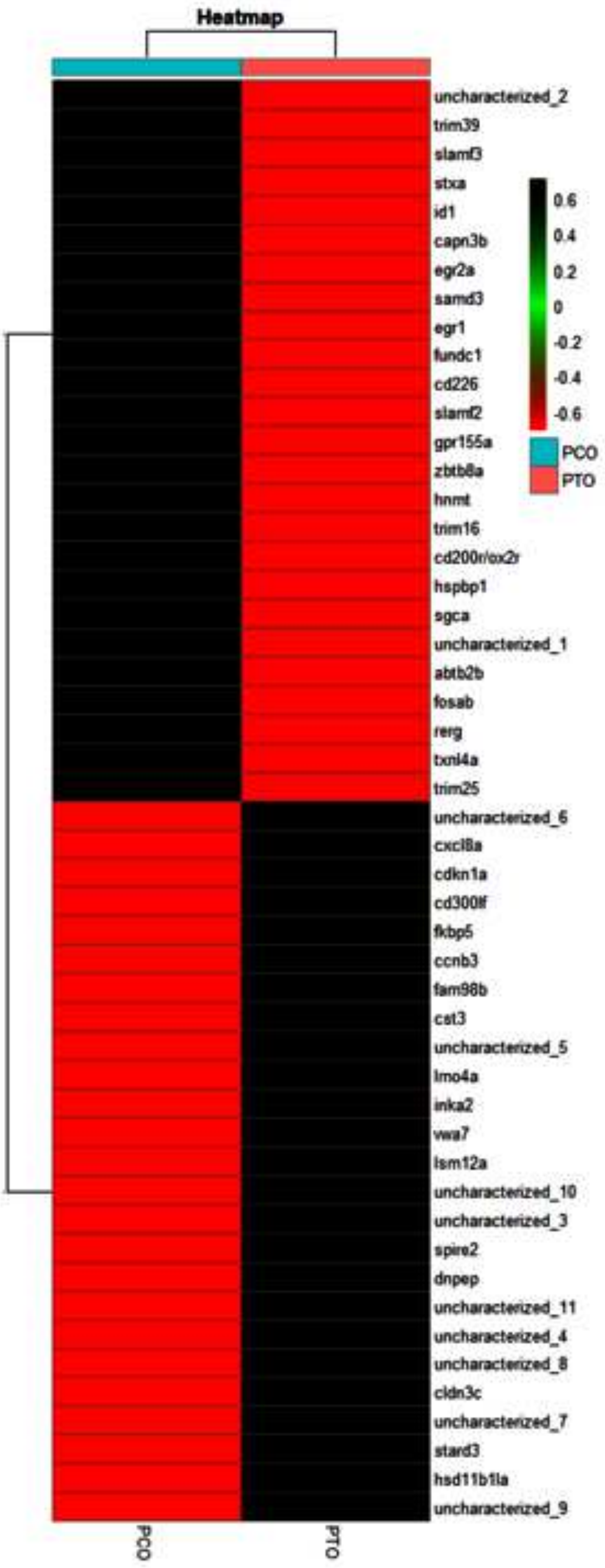

Figure 3

Click here to access/download;Figure;Fig3.tif

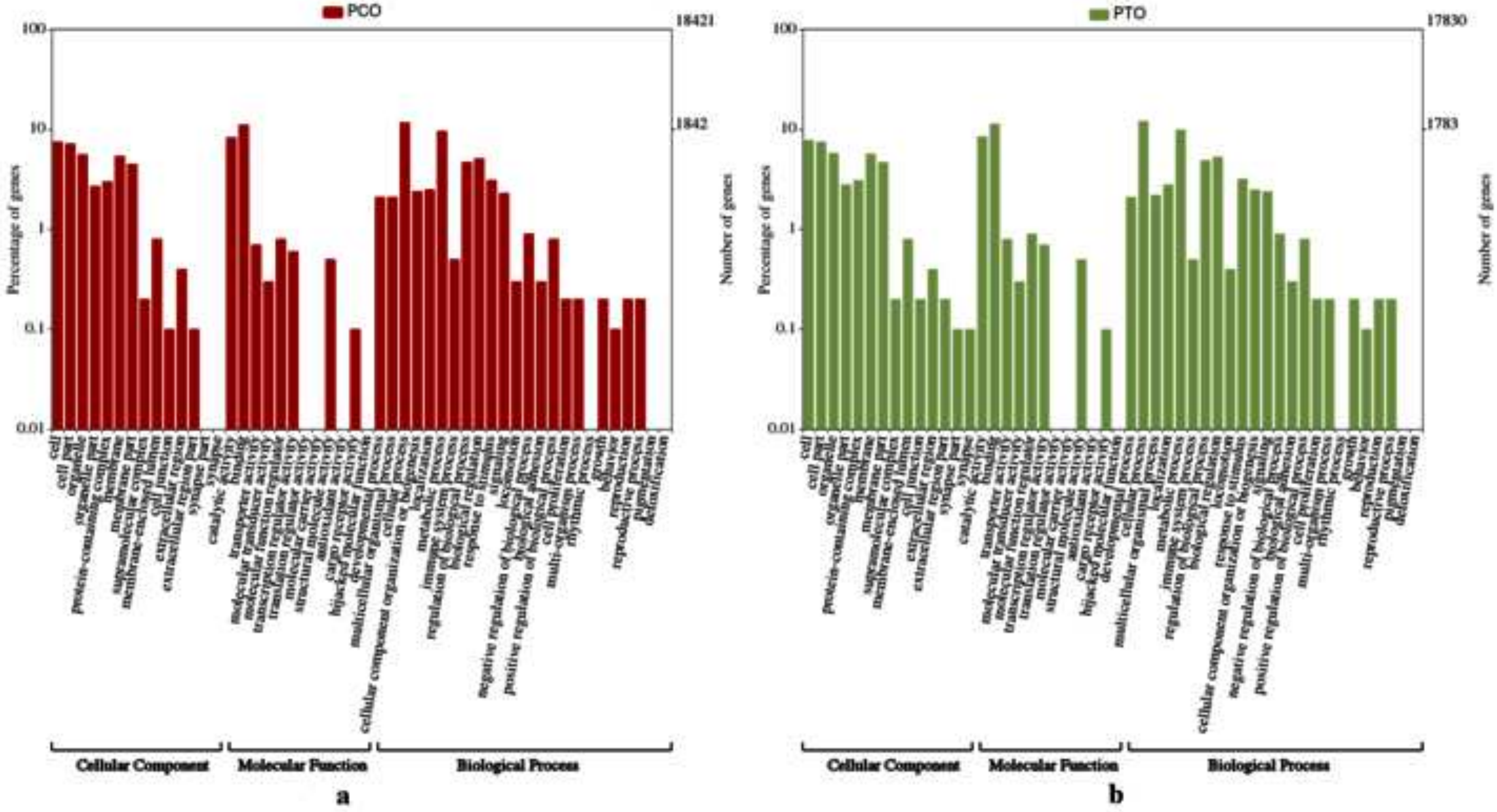

Tables

Click here to access/download
Other
Tables.docx
