# Supplementary material for: Effect of vegetable oil on ovarian steroidogenesis- A transcriptome approach to understand molecular mechanisms of hypothalamus pituitary and gonad axis (HPG) in Ompok bimaculatus
Source: PLoS One. 2024 Dec 4;19(12):e0309311. doi: 10.1371/journal.pone.0309311 (PMC11616869; doi:10.1371/journal.pone.0309311)
Supplement: Attachment — Submitted filename: comment.docx [file pone.0309311.s001.docx]

**Title:**

**Effect of vegetable oil on ovarian steroidogenesis- A transcriptome approach to understand 4 molecular mechanisms of hypothalamus pituitary and gonad axis in Ompok bimaculatus**

Dear editor, I want to express my appreciation for offering the opportunity to review this manuscript.

The Idea of the manuscript is novel and the methodology includes advanced assessment techniques. However, the manuscript still needs **a major revision.**

Thanks for your hard efforts. Please check the following comments

1- The abstract contains a lot of abbreviations that need to be rewritten fully (GSI, HIS, DEGs, HPG).
2- In the introduction section, the part between lines 58-66 needs to be rewritten to organize the aims of the study (please make the aims more direct)
3- In lines 76-77, what do you mean by; three tanks each in control and 77 treatment groups? Does that mean 3 tanks for each group?
4- What is the vegetable oil that has been used in this study? Mention its source and commercial data.
5- In lines 79-80 the mention of Table 1 here is not correct, it should be Table 3 instead. The table 1 should be in the results section. In addition, in the result section the author used Table 2 in describing the results of the fertilization rate and GSI that were described in Table 1, not Table 2.
6- In line 81 how did you know that the best treatment among these, i.e., T2
7- In line 88, what is the meaning of PTO and PCO?
8- How did you analyze the data statistically
9- In lines 155 – 156 please replace Table 2 with Table 1.
10- In line 181 Table 3 with Table 2.
11- In line 185 please replace the symptoms w3 and w6 with ω3 and ω6.
12- The discussion section needs to be rewritten (please do not repeat the results in the discussion)
