# Supplementary material for: Effect of vegetable oil on ovarian steroidogenesis- A transcriptome approach to understand molecular mechanisms of hypothalamus pituitary and gonad axis (HPG) in Ompok bimaculatus
Source: PLoS One. 2024 Dec 4;19(12):e0309311. doi: 10.1371/journal.pone.0309311 (PMC11616869; doi:10.1371/journal.pone.0309311)
Supplement: Attachment — Submitted filename: Response_to_Reviewers.docx [file pone.0309311.s003.docx]

**Response to Reviewers**

| **Reviewer Comments** | **Point-by-Point Response to Reviewers** |
| --- | --- |
| **Reviewer 1**  1- The abstract contains a lot of abbreviations that need to be rewritten fully (GSI, HIS, DEGs, HPG).  2- In the introduction section, the part between lines 58-66 needs to be rewritten to organize the aims of the study (please make the aims more direct)  3- In lines 76-77, what do you mean by; three tanks each in control and 77 treatment groups? Does that mean 3 tanks for each group?  4- What is the vegetable oil that has been used in this study? Mention its source and commercial data.  5- In lines 79-80 the mention of Table 1 here is not correct, it should be Table 3 instead. The table 1 should be in the results section. In addition, in the result section the author used Table 2 in describing the results of the fertilization rate and GSI that were described in Table 1, not Table 2.  6- In line 81 how did you know that the best treatment among these, i.e., T2  7- In line 88, what is the meaning of PTO and PCO?  8- How did you analyze the data statistically  9- In lines 155 – 156 please replace Table 2 with Table 1.  10- In line 181 Table 3 with Table 2.  11- In line 185 please replace the symptoms w3 and w6 with ω3 and ω6.  12- The discussion section needs to be rewritten (please do not repeat the results in the discussion) | 1- The abbreviations have been rewritten fully in the revised manuscript.  2- The paragraph has been rewritten to clearly state the aims.  3- Thank you for your comment. To clarify, by "three tanks each in control and treatment groups," we mean that there were a total of six tanks used in the experiment. Specifically, three tanks were designated for the control group and three separate tanks were designated for the treatment group. Each tank had a stocking density of 30 fish, ensuring that both control and treatment groups were adequately represented and environmental effects were minimized.  4- The source and commercial data for the vegetable oil has been mentioned properly in the revised manuscript line 86-92.  5- The table numbers have been rearranged properly.  6- T2 is identified as the best treatment for transcriptomics analysis based on several parameters observed in Table 2. The relative fecundity of T2 is significantly higher (743.39±2.50, 778.12±2.44, 749.16±3.6 eggs/g body weight) compared to other treatments and the control group. Additionally, T2 shows an improved fertilization rate (81.07±1.93, 79.00±2.06, 79.99±1.83%) and a notably higher Gonado Somatic Index (GSI) (24.46±1.07, 23.80±0.02, 23.25±0.40), indicating better reproductive health and development. These superior reproductive performance metrics make T2 the optimal candidate for a detailed transcriptomics analysis to understand the underlying molecular mechanisms. This has been properly mentioned in line 173-178 in the revised manuscript.  7- The PCO (Pabda Control Ovary) and PTO (Pabda Treated Ovary) has been properly mentioned in the revised manuscript,  8- The statistical analysis has been mentioned in the revised manuscript in line 168-174.  9- This has been corrected in the revised manuscript as suggested.  10- This has been corrected in the revised manuscript as suggested.  11- This has been corrected in the revised manuscript as suggested.  12- |
| **Reviewer 2**  Please modify Title; You have not addressed the source of vegetable oil in Abstract, and M&M. Please add this to The Title, Abstract, and M&M.  And in the title add HPG following Hypothalamus Pituitary Gonadal axis  Please modify "Jitendra Kumar Sundaray and Janmejay Parhi should be considered as joint corresponding author" to Jitendra Kumar Sundaray and Janmejay Parhi are equally responsible as corresponding Author.  Abstract:  Line 31 Please specify the source of vegetable oil  Line 33 Please indicate the full form of abbreviated words when the first appear in the text, i.e. GSI and HSI.  Introduction:  Line 42 change gonad to gonads  Line 43 change muscle to muscles  Line 50 change like to including  Line 56 change Fish to fish  Line 64 change So to therefore  M&M  Line 112 please revise and edit  Line 118 as above  Results  OK  Discussion:  Line 183 Please replace "depict" with an appropriate verb. Depict means represent by a drawing, painting, or other art form.  Again, Please indicate the source of vegetable oil.  Line 184 change "Several studies earlier describe" to Several earlier studies showed..  Line 184 Several studies earlier describe the beneficial effects of dietary lipid on  ovarian development (29-32). In which species?  Line 185 please correct omega symbol  Line 186 vitamins  Line 186 change gave a better result to gave a better understanding  Line 191 please revise. The writing style needs professional check.  Line 221 please revise  Line 267 can hence what?  Line 273 it can not | The necessary modifications have been made as suggested in the revised manuscript.  The necessary modifications have been made as suggested in the revised manuscript.  The source and commercial data for the vegetable oil has been mentioned properly in the revised manuscript line 86-92.  The necessary modifications have been made as suggested in the revised manuscript.  The necessary corrections have been incorporated.  The necessary corrections have been incorporated.  The necessary corrections have been incorporated.  The necessary corrections have been incorporated.  The necessary corrections have been incorporated.  The statement has been revised.  The statement has been revised.  The necessary corrections have been incorporated.  The necessary corrections have been incorporated in line 183, 184, 185, 186 and 191.  The statement has been revised.  This has been written clearly in the revised manuscript.  The necessary corrections have been incorporated. |
